# De-identification Methods for Open Health Data: The Case of the Heritage Health Prize Claims Dataset

**DOI:** 10.2196/jmir.2001

**Published:** 2012-02-27

**Authors:** Khaled El Emam, Luk Arbuckle, Gunes Koru, Benjamin Eze, Lisa Gaudette, Emilio Neri, Sean Rose, Jeremy Howard, Jonathan Gluck

**Affiliations:** ^1^Electronic Health Information LaboratoryCHEO Research Institute, Inc.Ottawa, ONCanada; ^2^Department of PaediatricsUniversity of OttawaOttawa, ONCanada; ^3^Department of Information SystemsUniversity of Maryland Baltimore CountyBaltimore, MDUnited States; ^4^Privacy Analytics, Inc.Ottawa, ONCanada; ^5^KaggleMelbourneAustralia; ^6^Heritage Provider NetworkNorthridge, CAUnited States

**Keywords:** Open data, de-identification, privacy

## Abstract

**Background:**

There are many benefits to open datasets. However, privacy concerns have hampered the widespread creation of open health data. There is a dearth of documented methods and case studies for the creation of public-use health data. We describe a new methodology for creating a longitudinal public health dataset in the context of the Heritage Health Prize (HHP). The HHP is a global data mining competition to predict, by using claims data, the number of days patients will be hospitalized in a subsequent year. The winner will be the team or individual with the most accurate model past a threshold accuracy, and will receive a US $3 million cash prize. HHP began on April 4, 2011, and ends on April 3, 2013.

**Objective:**

To de-identify the claims data used in the HHP competition and ensure that it meets the requirements in the US Health Insurance Portability and Accountability Act (HIPAA) Privacy Rule.

**Methods:**

We defined a threshold risk consistent with the HIPAA Privacy Rule Safe Harbor standard for disclosing the competition dataset. Three plausible re-identification attacks that can be executed on these data were identified. For each attack the re-identification probability was evaluated. If it was deemed too high then a new de-identification algorithm was applied to reduce the risk to an acceptable level. We performed an actual evaluation of re-identification risk using simulated attacks and matching experiments to confirm the results of the de-identification and to test sensitivity to assumptions. The main metric used to evaluate re-identification risk was the probability that a record in the HHP data can be re-identified given an attempted attack.

**Results:**

An evaluation of the de-identified dataset estimated that the probability of re-identifying an individual was .0084, below the .05 probability threshold specified for the competition. The risk was robust to violations of our initial assumptions.

**Conclusions:**

It was possible to ensure that the probability of re-identification for a large longitudinal dataset was acceptably low when it was released for a global user community in support of an analytics competition. This is an example of, and methodology for, achieving open data principles for longitudinal health data.

## Introduction

Creating open data is considered an important goal in the research community. Open data is said to ensure accountability in research by allowing others access to researchers’ data and methods [1-4]. Having research data available to peers and the public helps to ensure that reported study results are valid and protects against faulty data [1-8]. Another asset of open data in research is that it allows researchers to build on the work of others more efficiently and helps to speed the progress of science [2,3,5]. To build on previous discoveries, there must be trust in the validity of prior research. Openness of research methods, as well as of raw data, facilitates trust between researchers and with the public [2]. Having research data available to other researchers allows for secondary analyses that expand the usefulness of datasets and the resulting knowledge gained [1,3-5]. Connected to this is the decrease in the burden on research participants through the reuse of existing research data and the decrease in the cost of data collection [1,3,5].

Although there is some evidence that sharing raw research data increases the citation rate of research papers [9], researchers do have concerns about open data, including the privacy of research participants, which could prevent them from sharing data [1,3,4,6,8,10-12]. Research participants put their trust in the research team to protect their privacy and keep their information confidential [3,4,8,12,13].

There is a dearth of articles documenting methods for the creation of open health data that specifically address these privacy concerns. We provide a case study of de-identifying a health dataset for public release in the context of the Heritage Health Prize (HHP).

### The Heritage Health Prize

In April 2011 the Heritage Provider Network (HPN), a health maintenance organization based in California, launched the largest public health analytics competition to date: the HHP [14]. The objective of the competition is to construct a model to predict the number of days a patient will be hospitalized in the following year, by using the current and previous years’ claims data. The core dataset consists of 3 years of de-identified HPN data on 113,000 patients. At the time of writing there were 1347 entrants in the competition and close to 10,000 entries. The patient data are provided to all entrants through a download on the competition website. The individual or team that develops the most accurate prediction model past a certain accuracy threshold after the 2-year competition period gets a US $3 million cash prize, or a $0.5 million prize for the most accurate model if no entrant beats the accuracy threshold.

The public disclosure of health data for the purposes of attracting data analysts from around the globe to solve complex problems or to bring rapid advances to a field is not new. Table 1 [15-18] summarizes three recent health competitions that made data publicly available. However, the privacy of patients is an important consideration when publicly disclosing a large health dataset accessible with few restrictions. In particular, there is a risk that patients in the competition dataset can be re-identified by an adversary. Re-identification can potentially harm these patients, from social and psychological harm, to financial harm by affecting their employability or insurability.

In the United States there is no legislative requirement to obtain patient consent to disclose health information if the data are deemed de-identified. The Health Insurance Portability and Accountability Act (HIPAA) Privacy Rule provides some definitions and standards for the de-identification of health data. Therefore, a credible claim must be made that the data are indeed de-identified according to one of those standards to allow their disclosure for the HHP without obtaining patient consent.

**Table 1:** Recent examples of public releases of health data for the purpose of competitions.

Competition	Objective
Predict HIV Progression [15]	Finding markers in the human immunodeficiency DNA sequence that predict a change in the severity of the infection
INFORMS data mining contest [16]	Predicting hospitalization outcomes of transfer and death
Practice Fusion medical research data [17,18]	Developing an application to manage patients with a focus on chronic diseases

### De-identification of the HHP Data

We describe how the HHP data were de-identified for the competition to (1) make the data publicly available, and (2) meet the requirements of the HIPAA Privacy Rule. Only one previous study explained the methods for de-identifying public-use health data files; however, it considered risks to Canadian patients and did not involve longitudinal data [19]. The main objective of the de-identification was to protect the identity of the patients.

The contributions of this work are (1) a description of how we measured re-identification risk for the public release of a large health dataset in the United States (which can be a useful example for other open government and open data initiatives and programs in the United States [20]), including the development of new risk measurement techniques, (2) an extension of an existing algorithm developed for cross-sectional data, optimal lattice anonymization, with new methods to efficiently de-identify longitudinal data, and (3) a description of an approach for using simulated attacks to evaluate longitudinal data de-identification algorithms and an illustration of its use on the HHP dataset. Our results demonstrate that it is possible to publicly disclose longitudinal health data with strong guarantees of privacy, as defined in HIPAA. Furthermore, the methods described here can serve as a template for other open competitions and the creation of open health datasets.

## Methods

The competition data consist of 3 years’ worth of demographic and claims data. For year 1 and year 2, the number of days of hospitalization in the subsequent year is also included. The claims data represent the predictors, and the number of days of hospitalization is the outcome. These data are used for training prediction models. Entrants use the year-3 claims data to predict the number of days of hospitalization for year 4, and the competition will be judged on the accuracy of that year-4 prediction. Therefore, entrants download the data for years 1–3, to predict days of hospitalization for year 4.

Managing the re-identification risk for the competition dataset consists of a combination of technical and legal measures. These measures are described in the following section.

### Definitions

#### Quasi-identifiers

*Quasi-identifiers* are variables that represent the background knowledge about patients in the competition data that an adversary could use for re-identification. If an adversary does not have certain background knowledge, then those variables cannot be quasi-identifiers. General examples of quasi-identifiers are sex, date of birth or age, location information (such as zip codes), language spoken at home, and ethnic origin.

#### Equivalence Classes

All records that share the same quasi-identifier values are called an *equivalence class*. For example, all the records in a dataset about 17-year-old males admitted on January 1, 2008 are an equivalence class. Equivalence class sizes for a quasi-identifier (such as age) could potentially change during de-identification. For example, there may be 3 records for 17-year-old males admitted on January 1, 2008. When the age is recoded to a 5-year interval, then there may be 8 records for males between 16 and 20 years old admitted on January 1, 2008. In general there is a trade-off between the level of detail provided for a quasi-identifier and the size of the corresponding equivalence classes, with more detail being associated with smaller equivalence classes.

#### Identity Versus Attribute Disclosure

Two kinds of disclosure are of concern. The first occurs when an adversary can assign an identity to a record in the disclosed dataset. For example, if the adversary is able to determine that record number 7 belongs to patient Alice Smith, then this is called *identity disclosure*. The second type of disclosure happens when an adversary learns something new about a patient in the database without knowing which specific record belongs to that patient. For example, if all 20-year-old female patients in the disclosed database who live in a particular county have a particular diagnosis, then an adversary does not need to know which record belongs to 20-year-old Alice Smith, if she lives in that county, to know that she has that particular diagnosis. This is called *attribute disclosure*.

All known re-identification attacks of personal information that have actually occurred have been identity disclosures [21]. Furthermore, the HIPAA Privacy Rule is concerned only with protecting against identity disclosure. Consequently, identity disclosure was the primary risk that needed to be addressed. We therefore focused solely on identity disclosure for the purpose of de-identification.

### Dataset

The claims dataset consists of two tables that include the fields shown in Table 2 and Table 3 [22,23]. The records in both tables are linked through the MemberID field. The *patients* table has only 1 record per patient. The *claims* table contains records for all of the patient claims included in the dataset. Patients have different numbers of claims over the 3-year period.

The quasi-identifiers included in the dataset (indicated in Table 2 and Table 3) are the variables that we believed could be used by an adversary for a re-identification attack. The justification for this selection of quasi-identifiers will become evident below when we discuss the plausible re-identification attacks that could be made on these data.

**Table 2:** Description of the fields in the patients data table.

Field	Description
MemberID	Unique identifier for the patient
Age^a^	Age in years at the time of the first claim in year 1
Sex^a^	Patient’s sex
DaysInHospital Y2^a^	Total number of days the patient was hospitalized in year 2
DaysInHospital Y3^a^	Total number of days the patient was hospitalized in year 3

^a^ Quasi-identifier.

**Table 3:** Description of the fields for the claims data table.

Field	Description
MemberID	Unique identifier for the patient
ProviderID	Unique identifier for the responsible provider giving care
Vendor	Unique identifier for the vendor providing the service
PCP	Unique identifier for the primary care provider
Year	Indicator of claim year (year 1, year 2, or year 3)
Specialty^a^	Specialty of provider
PlaceOfService^a^	Place of service
CPTCode^a^	CPT^b^ code: these codes provide a means to accurately describe medical, surgical, and diagnostic services, are used for processing claims and for medical review, and are the national coding standard under HIPAA^c^
LOS^a^	Length of stay in hospital
DSFC^a^	Number of days since first claim computed from the first claim for that patient for each year
PayDelay	Number of days of delay between date of service and date of payment of the claim
Diagnosis^a^	ICD-9-CM^d^ code

^a^ Quasi-identifier.

^b^ Current Procedural Terminology [22].

^c^ Health Insurance Portability and Accountability Act.

^d^ *International Classification of Diseases*, 9th revision, Clinical Modification [23]

### Preprocessing of Claims Data

We preprocessed the data to apply some basic de-identification steps before assessing any quantitative re-identification risk.

#### Creating Pseudonyms

The MemberID, ProviderID, Vendor, and PCP fields were converted to irreversible pseudonyms [24], since they would otherwise be considered direct identifiers. These ID values are used during the provision of care and therefore are generally known. Consequently, these direct identifiers could potentially be exploited for financial gain, and were therefore pseudonymized. For example, the original IDs could be used to identify individual providers and the number and type of procedures that they perform.

#### Top-Coding

Quantitative values that are considered uncommonly high are often limited to an upper bound, a procedure called *top-coding*. Such extreme values make individuals more unusual (or make them stand out) in the population and can be revealing by themselves or used to infer other characteristics about the patients that should be protected.

A commonly used heuristic for top-coding is to have a cut-off at the 99.5th percentile [25]. To err on the conservative side, we top-coded the PayDelay variable and the DaysInHospital variable at the 99th percentile. Extreme values on PayDelay could indicate procedures that are more expensive and for which it would take an unusually long time to pay (and hence the procedure could be inferred from the PayDelay value). Extreme values for DaysInHospital could cause patients to stand out because they have stayed exceedingly long in hospital.

#### Truncation of Claims

While it is not likely that an adversary would know the exact number of claims that an individual patient would have, it is plausible for an adversary to know whether an individual patient has had an abnormally large number of claims. For example, a patient may have 300 claims a year and be the only one in the population with more than 200 claims. Adversaries who know that their 50-year-old neighbor has had an unusually high number of hospital procedures could correctly guess that this extreme outlier is their neighbor.

We therefore truncated the number of claims per patient at the 95th percentile. To decide which claims to truncate we assigned each claim a score, and deleted claims with the highest scores from the dataset. A description of the scoring method is provided in Multimedia Appendix 1.

The truncation of claims was different from the censoring method that has been described in previous research for diagnosis codes [26]. The censoring method collapses repeating codes and then uses suppression to ensure a minimal number of patients have the same code. In our case we did not collapse similar claims, and our focus was on full claims that contained multiple pieces of information (as summarized in Table 3), as opposed to just diagnosis codes.

#### Removal of High-Risk Patients and Claims

Patients who were considered to be high risk were removed from the dataset to avoid the chance that their disease, condition, or procedure could be inferred from patterns in the data. These patients had *International Classification of Diseases*, 9th revision, clinical modification (ICD-9-CM) or Current Procedural Terminology (CPT) codes that represent highly stigmatized conditions, and conditions that patients who lock their records (through consent directives) tend to have and want to conceal—for example, patients with human immunodeficiency virus infection, those who have had abortions, and patients with rare and visible diseases and conditions [27]. For those individuals HPN deemed that the only acceptable risk of re-identification was zero. The criteria used to remove patients are listed in Multimedia Appendix 1.

As Multimedia Appendix 1 shows, the patients who were removed during this step were different on several factors from the rest of the patient population: (1) their length of stay (LOS) in hospital tended to be longer, (2) they tended to be older, (3) the interval between their claims was longer, and (4) they tended to have more claims. This suggested that they had more serious chronic conditions than the rest of the population dataset. On the other factors there was little or no difference.

#### Suppression of Provider, Vendor, and PCP Identifiers

Providers could have patterns of treatment that make them stand out. An adversarial analysis by an independent party of a prerelease version of the HHP dataset noted how information about providers could potentially be used to predict the hospitals where procedures were performed (A Narayanan, unpublished data, 2011). Knowledge of the treating hospital would increase the risk of re-identification for the patients.

These patterns of treatment consisted of 4 quasi-identifiers: the place of service, specialty, CPT code, and diagnosis code. For example, a provider could be the only one with a particular specialty in a specific place of service who performed procedures on patients with a particular diagnosis. In cases where it was estimated from the HHP data that there were fewer than 20 providers with the same pattern in the HPN system, the provider ID was suppressed for those records. The choice of 20 is justified below in the section outlining thresholds. The estimation method used is described elsewhere [28,29]. A similar process was followed for vendor and primary care provider IDs.

### Facts and Assumptions About Re-identification Threats

To understand the type of de-identification required to protect patients, we first had to determine the threats that could exist for the duration of the competition. The following are the key facts and assumptions of the threat modeling used:

- Fact: The dataset that was being released for the HHP consisted of a small sample of all HPN patients.
- Fact: All entrants in the competition had to sign (or click through) an agreement saying that they would not attempt to re-identify patients in the dataset, contact patients, or link the HHP data with other datasets that would add demographic, socioeconomic, or clinical data about the patients (where such data could make the risk of re-identification much higher).
- Assumption: It would not be possible for an adversary to know whether the record for a particular patient was in the HHP dataset. If an adversary made a guess, it would be equal to the sampling fraction. Most patients would themselves not know whether they were members of HPN, and therefore the most realistic sampling fraction to use would be from the population of counties in California covered by HPN. However, to err on the conservative side, we assumed that an adversary would know whether a patient was a member of HPN in our calculations of re-identification risk.
- Assumption: An adversary would have background information about only a subset of the claims of a patient in the dataset. For example, if a patient had 100 claims, we did not deem it plausible for the adversary to know the exact information in all of those 100 claims and to use that information for re-identification purposes. Rather, we assumed the adversary would have information about only a subset of these claims. This has previously been referred to as the *power* of the adversary, and various methods have been used to account for power when de-identifying transactional data [30-32].

These facts and assumptions shaped how we conceptualized re-identification risk and which kinds of attacks we considered plausible for this dataset.

### Attacks on the Data

We examined plausible attacks on the data as described below, and for each one we will discuss how we measured and managed the re-identification risks.

One important distinction to make at the outset pertains to subcontractors (eg, insurers, laboratories, or pharmacists) and employees of HPN, versus the entrants. Subcontractors process patient data during the regular provision of care and will have a large amount of information about the patients in the competition that can potentially be used for re-identification. However, HPN has contracts with these subcontractors and there are already mechanisms in place to enforce these agreements. In such a case, reliance on existing legal methods to protect against re-identification by subcontractors was deemed sufficient.

On the other hand, entrants in the competition could come from many countries in the world. Even though entrants had to agree to a certain set of rules, enforcement of the rules globally poses a practical challenge.

Therefore, we assumed that an adversary would be one of the entrants who has obtained the HHP data (1) by registering for the competition, or (2) through a data leak (deliberate, accidental, or malicious) from a legitimate entrant. Furthermore, it would not be prudent to assume that the adversary would adhere to conditions on other public or semipublic databases to which they have gained access. In such a case, we needed technical methods that provide stronger guarantees that the probability of re-identification is low.

#### Attack 1: The Nosey Neighbor Adversary

Under this attack, the adversary would be an individual who (1) would be trying to re-identify a target individual who was an HPN patient (a specific individual, such as a neighbor or a famous person) or any individual who was known to the adversary to be an HPN patient (an arbitrary individual selected at random), (2) would not know whether the target individual was in the dataset, and (3) would have some basic background information about the target patient in terms of the patient’s demographics and information about some of the patient’s claims.

The adversary could be a patient’s neighbor, coworker, relative, or ex-spouse, or the target individual could be a famous person whose basic demographics and perhaps some of whose treatment information would be publicly known. There are known examples of this kind of attack. In one case a researcher re-identified the insurance claim transactions of the Governor of Massachusetts [33]. In another example, a neighbor was re-identified in a hospital prescription database that was going to be disclosed to a commercial data broker [34].

Under this type of attack, the risk metric would be the probability that an individual can be correctly re-identified. The probability of an individual being re-identified using this attack is the reciprocal of the equivalence class size in the HPN member population (from which the competition dataset is derived) [28].

**Figure 1:**
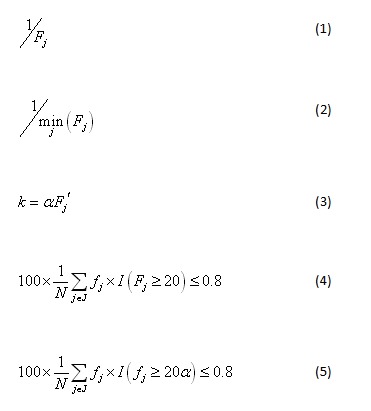
Equations describing how re-identification risk was measured.

For any patient in an equivalence class *j*, the probability of re-identification was defined as equation 1 (Figure 1), where *F_j_* is the equivalence class size in the HPN patient population. Since we did not know which record might be attacked, we used the record with the highest risk as a risk measure for the whole file (equation 2, Figure 1).

#### Attack 2: Matching With the Voter Registration List

In California it is possible to obtain the voter registration list [35]. The voter registration list contains the date of birth and gender of the voter. We did not include the full date of birth in the HHP dataset, but we did include a generalization of the date of birth to a 10-year interval. Even though there are restrictions on what a California voter registration list can be used for [35], an adversary could potentially match the HHP dataset with the voter list to re-identify patients. In such a case, the appropriate re-identification risk metric would be the proportion of individuals that could be re-identified in the HHP dataset (this metric is also known as marketer risk [29]).

It has been shown that managing the risk in equation 2 also manages marketer risk [29]. And, because the equivalence classes in the voter registration list are the same size as or larger than the equivalence classes in the HPN population, if we could protect against attack 1, then we would automatically protect against attack 2.

#### Attack 3: Matching With the State Inpatient Database

In the United States, 48 states collect data on inpatients [36], and 26 states make their hospital discharge data available through the Agency for Healthcare Research and Quality (AHRQ) [37]. These data can be purchased for the purposes of research or another approved use. These datasets are referred to as the State Inpatient Database (SID).

An adversary could potentially match the competition dataset with the SID data to discover something new about the individuals in the dataset. For example, if an individual were able to match the HHP records with the SID records, then the adversary could discover the exact month and year of birth of patients and their detailed diagnosis codes and procedures, even if we generalized them in the HHP data release (since these fields are included in the SID). Furthermore, the SID contains race information, which could be added to the HHP dataset after matching. This would provide more detailed information than was disclosed in the HHP dataset and would therefore raise the re-identification risk for any correctly matched patients.

Note that not all patients in the HHP dataset were hospitalized. Some may, for example, have been seen in an outpatient clinic. Therefore, by definition only a subset of the HHP dataset could be matched with the SID.

For this attack, the re-identification risk metric would be the proportion of individuals that could be matched between the HHP and the SID datasets. This can be measured using the marketer risk metric [31].

Since the SID covers all hospital discharges in California, the equivalence class sizes for hospitalized patients in the HPN population were equal to or smaller than the SID equivalence classes for those patients. This means that if we managed the risk in equation 2 for attack 1, we would also manage the risk for attack 3.

#### Summary of Re-identification Risks from Attacks

Based on the above analysis of the various possible attacks, if the re-identification risk from attack 1 could be managed, then the risks from all of the other attacks would also be managed. Below we describe the algorithm used in this study to manage the risk from attack 1. Additionally, during the empirical evaluation component of our study, we measured the re-identification risks from attacks 1 to 3 to confirm that the re-identification risks for all three attacks were acceptably low.

### Methods for the De-identification of the HHP Dataset

We used an automated algorithm to de-identify the dataset through generalization. Our base automated de-identification algorithm was OLA [38]. OLA provides a globally optimal solution and has been shown to have good performance on real health datasets [38]. It is a k-anonymity algorithm. The k-anonymity criterion is one of the most common ways to de-identify a dataset [38-42] and can be used to manage the probability of re-identification due to identity disclosure [28]. OLA has been designed to work only on cross-sectional data. As we describe further below, we have extended this algorithm to de-identify longitudinal data. We refer to our extended algorithm as longitudinal OLA (LOLA).

#### Base Algorithm

We will provide a brief overview of how LOLA works and its parameters, and then explain how we modified these parameters for the de-identification of the longitudinal HHP dataset.

##### Input

LOLA has two inputs. The first is the *k* value, which indicates the maximum amount of re-identification risk the data custodian is willing to take, and this determines the amount of de-identification that will be applied. The *k* value is the minimum size of an equivalence class. This means that the maximum probability of a record being correctly re-identified is given by 1/*k* (this is the risk threshold). LOLA’s second input pertains to the percentage of records that have a risk higher than the risk threshold: the *MaxSup* parameter.

In our case we defined *k* = *f_j_*, where the *f_j_* value is the minimum equivalence class size in the HHP dataset. Since the risk we wanted to manage was 1/*F_j_*, which was based on the equivalence class sizes in the HPN member population, we made the large sample assumption that *f_j_* = α*F_j_*, where α is the sampling fraction and *F_j_* is the smallest equivalence class size in the HPN population. Therefore, we defined equation 3 (Figure 1).

For example, if we had set *F_j_* = 20 and a 20% sampling fraction was used, then the *k* value for LOLA would have been 4.

Note that in practice more sophisticated methods for estimating *f_j_* would be used as described elsewhere [28,29], especially for small sampling fractions. In our case, we used an estimator based on the truncated Poisson distribution [28].

##### Generalization

**Figure 2:**
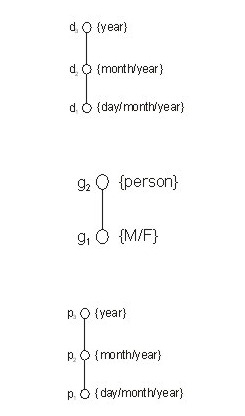
The three domain generalization hierarchies for the 3 quasi-identifiers: date of birth (d), gender (g), and visit date (p).

A key step in LOLA is generalization. Generalization reduces the precision in the data. As a simple example, a patient’s date of birth can be generalized to the month and year of birth, to the year of birth, or to a 5-year interval. Allowable generalizations are specified in generalization hierarchies. Let us consider an example dataset with only 3 quasi-identifiers: date of birth (d), gender (g), and date of visit (p). Figure 2 shows the domain generalization hierarchies for these quasi-identifiers. These hierarchies describe how the precision of each quasi-identifier can be reduced during generalization.

**Figure 3:**
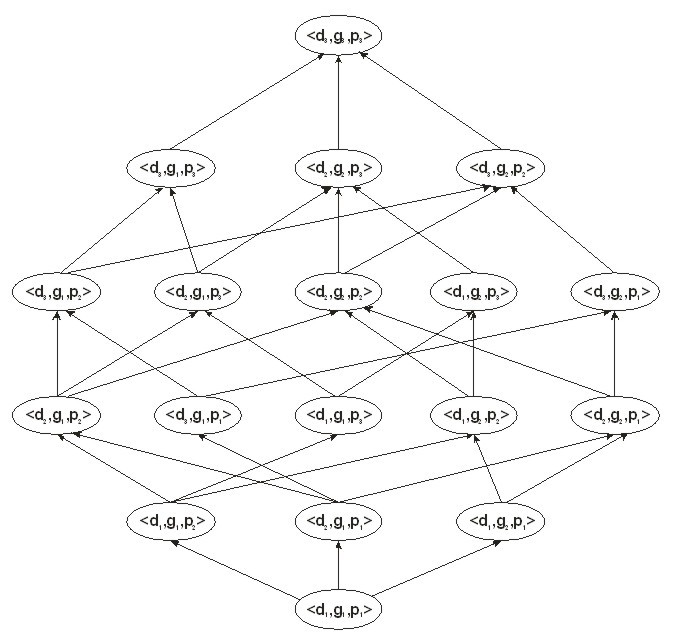
A lattice showing the possible generalizations of the 3 quasi-identifiers: date of birth (d), gender (g), and visit date (p).

All of the possible generalizations can be expressed in the form of a lattice as shown in Figure 3. In this lattice each possible generalization is represented by a node starting from the original dataset at the lowest node, <d_1_,g_1_,p_1_>. As one moves up the lattice, the quasi-identifiers are generalized. For example, node <d_2_,g_1_,p_1_> has the date of birth generalized to month and year. The objective of LOLA is to efficiently find the best generalization solution (node) in that lattice. The best node meets two criteria: (1) the proportion of records that are considered to have a high probability of re-identification is less than or equal to *MaxSup*, and (2) the best node has the smallest amount of information loss.

After efficiently evaluating the nodes in the lattice, LOLA identifies the candidate nodes that meet criterion 1 above. Out of the candidate nodes, LOLA then chooses the node with the smallest information loss among the candidate nodes, and this meets criterion 2. Information loss is measured in terms of a general entropy metric, which was found to have properties superior to those of other commonly used metrics in the literature [38].

#### Acceptable Re-identification Risk

In the United States, the HIPAA Privacy Rule Safe Harbor de-identification standard was conceptualized using population uniqueness of individuals as the measure of risk, as documented in the responses to comments by the Department of Health and Human Services [43,44]. The population uniqueness standard means that individuals who are the only one in their equivalence class are at high risk, but if there is more than one person in the equivalence class then they are not considered at a high risk of re-identification. The designers of Safe Harbor considered very small” risk in terms of the percentage of the population that is unique. Studies have shown that in datasets that meet the Safe Harbor de-identification standard, 0.04% of the population is unique [45,46]. HPN wanted to ensure that the risk exposure for the HHP dataset was equal to or less than the Safe Harbor risk exposure. Risk exposure is defined as *loss* × *probability*.

Risk exposure only comes from the records that have an unacceptably high probability of re-identification. In our case, the *loss* pertained to the number of individuals who could be re-identified (ie, individuals at a high risk of re-identification), and *probability* meant the probability of re-identification. Therefore, for a dataset with *N* records, the maximum risk exposure under Safe Harbor would be 0.004 × *N* × 1.

A uniqueness threshold would be considered quite high by most standards (for example, see [13,47-60]). HPN decided that a probability of re-identification for *a record* in the HHP dataset of .05 was an acceptable risk. The choice of this value was informed by two precedents. The Center for Medicaid & Medicare Services has created public-use files containing claims data from a sample of beneficiaries while ensuring that the probability of an individual being re-identified was less than .1 [61]. An analysis to prepare a public discharge abstract dataset from Canadian hospitals used a threshold probability of .05 [19]. The value chosen by HPN erred on the more conservative side of this range.

This means that, if the probability was equal to or lower than .05, then the data would be acceptable for release. To ensure a risk level that low we needed to ensure that *F_j_* ≥ 20 for all *j* (ie, *F_j_* = 20 in equation 3).

If we revisit our definition of *k* in equation 3, we would have *k* = 30α, since this threshold translates to a maximum equivalence class size of 20 in the population.

To retain the same level of maximum risk exposure as Safe Harbor with our proposed .05 probability threshold, we could accept only 0.8% of the records to have a probability that was higher than the .05 threshold for the same value of *N* (ie, .05 × 0.008 × *N* = 1 × 0.004 × *N*). The 0.8% value then represented the *MaxSup* that was used in the LOLA algorithm.

Therefore, if the condition in equation 4 (Figure 1) was met, then we considered the risk acceptable. In equation 4, *I*(·) was the indicator function. Making the large sample simplification resulted in equation 5 (Figure 1), although, as noted above, better estimates of *F_j_* could be used for small samples [28,29].

#### Generalization Hierarchies for the HHP Dataset

As Table 4 shows, 4 quasi-identifiers had generalization hierarchies: Age, DaysInHospital, LOS, and DSFC. These were the ones we used to construct the lattice for the application of LOLA. For the remaining quasi-identifiers there was only one level of predetermined generalization.

Each claim had up to 4 diagnosis codes. These were converted into 2 values. The ICD-9-CM diagnosis codes were generalized into 45 primary condition groups, which have been determined to be good predictors of mortality [62]. We also created a categorized comorbidity score (Charlson index) [63,64]. The CPT codes were generalized to a higher code in the CPT hierarchy. In consultation with clinical experts, we also grouped values within the Specialty and PlaceOfService variables. All groupings are described in Multimedia Appendix 1. Table 4 summarizes all of the generalization hierarchies used.

**Table 4:** Description of the generalization hierarchies for the quasi-identifiers.

Quasi-identifier	Description
Age	Years → 5-year interval; 80+ → 10-year interval; 80+ → 20-year interval; 80+
Sex	no change
DaysInHospital Y2/Y3	Days → days to 2 weeks; >2 weeks → days to 1 week; 1–2 weeks; >2 weeks
Specialty	Original specialty → grouped specialty (see Multimedia Appendix 1)
PlaceOfService	Original place of service → grouped place of service (see Multimedia Appendix 1)
CPTCode^a^	Original CPT code → grouped CPT code
LOS^b^	Days → days up to 6 days, weeks afterward → days up to 6 days; (1–2] weeks; (2–4] weeks; (4–8] weeks; (8–12 weeks]; (12–26] weeks; 26+ weeks → <1 week; (1–2] weeks; (2–4] weeks; (4–8] weeks; (8–12 weeks]; (12–26] weeks; 26+ weeks → <4 weeks; (4–8] weeks; (8–12 weeks]; (12–26] weeks; 26+ weeks
DSFC^c^	Days → weeks → 2 weeks → months
Diagnosis	ICD-9-CM^d^ code → primary condition group (see Multimedia Appendix 1)

^a^ Current Procedural Terminology.

^b^ Length of stay in hospital.

^c^ Days since first claim.

^d^ *International Classification of Diseases*, 9th revision, Clinical Modification.

#### Adversary Knowledge and Power

The *power* of the adversary reflects the number of claims that the adversary would have background information about, and it pertains to the claims data and not to the basic information about the patients (such as their demographics). We denoted the power of the adversary as *p*. If each claim had only 1 quasi-identifier, then *p* would mean that the adversary had knowledge about the value for that quasi-identifier in *p* claims. With more than 1 quasi-identifier in each claim, the adversary would know each of the quasi-identifier values for *p* claims.

Previous research that considered the power of the adversary always assumed that the power is fixed for all patients [30-32,65,66]. However, intuitively it makes sense that the adversary would have different amounts of background knowledge, or power, for different patients. For example, everything else being equal, it is easier to have background information about a patient with a large number of claims than about a patient with few claims. Therefore, we would expect power to increase monotonically with the number of claims that a patient has.

Also, it is likely that certain pieces of background information are more easily knowable than others by an adversary, making it necessary to treat the quasi-identifiers separately when it comes to computing the power of an adversary. For example, it would be easier to know a diagnosis value for patients with chronic conditions whose diagnoses keep repeating across claims. In such a case, if the adversary knew the information in 1 claim, then it would be easier to predict the information in other claims, increasing the amount of background knowledge that the adversary can have. In this case the diversity of values on a quasi-identifier across a patient’s claims becomes an important consideration. Therefore, we expect the power of an adversary to decrease monotonically with the diversity of values on the quasi-identifiers.

As Multimedia Appendix 1 shows, we found that the correlations among the diversity of quasi-identifiers tend to be small to moderate. For example, the correlation between the diversity of Specialty and LOS was .08. This means that there is a very weak relation between the diversity in these two variables. On the other hand, the correlation between the diversity of Specialty and CPTCode was .4 and that between LOS and DSFC was –.44. In the former case it means that the more varied the specialty of the treating physician, the more varied the procedures. In the latter case, the more varied the LOS, the less varied the time between claims. These relationships make sense, but they also suggest that it would be more appropriate to compute a power for each quasi-identifier separately, because diversity is not uniform across quasi-identifiers for the same patient.

We defined the power for a particular individual in the data and for a particular quasi-identifier as *p_ih_*, where *i* represents the individual and *h* represents the quasi-identifier. In Multimedia Appendix 1 we present a method for computing a value for *p_ih_* that takes into account the number of claims and diversity. We also set the maximum power *p_m_* as max(*p_ih_*) = 5. This means that, for any single patient, the maximum number of values (claims) that an adversary would have is 5 for each quasi-identifier. For instance, if there are 2 quasi-identifiers, then the adversary can have a maximum of 10 pieces of information on the patient. In our empirical evaluation we assessed the sensitivity of our results to this value.

We made two assumptions about the knowledge of the adversary: (1) the adversary would not know which values on the quasi-identifiers were in the same claim (the *inexact knowledge* assumption), and (2) the adversary would not know the exact order of the claims (the *inexact order* assumption) beyond what is revealed through the DSFC quasi-identifier, which is consistent with other models of transactional data in the disclosure control literature [30-32,65,66]. However, we did test the sensitivity of our results to these assumptions in our empirical evaluation.

#### Node Computation

As noted earlier, the LOLA algorithm performs an efficient search through the lattice. During this search it needs to evaluate the percentage of records that are high risk for some of the nodes in the lattice. This is called *node evaluation*.

It would have been computationally very expensive for us to evaluate all combinations of *p_ih_* values for each quasi-identifier. For example, computing all combinations of 5 values from, say, 100 claims would have required more than 75 million computations of risk. Since these 5 combinations would be different for each patient, this would need to be repeated tens of thousands of times.

Therefore, we used a hierarchical bootstrapping approach [67]. Here we sampled 10,000 patients with replacement, and for each sampled record we selected *p_ih_* quasi-identifier values across the claims without replacement. We then computed the proportion of patients who were at high risk in each iteration and took the mean across 1000 iterations. If the mean number of patients who were flagged as high risk was greater than *MaxSup*, then we did not consider the node to be a candidate solution (see equation 5, Figure 1). Multimedia Appendix 1 provides a complete description of the node computation.

### Empirical Evaluation

After the de-identification of the dataset using LOLA, we wanted to empirically evaluate whether the risks from the three plausible attacks were appropriately managed. Hence, we performed an empirical evaluation.

#### Attack 1

To evaluate the actual probability of re-identification under this attack, we developed a separate attack program that would simulate exactly what an adversary would do. This program was developed by an independent programmer not involved in the development and application of LOLA described above.

The simulated attack assumed that an adversary would choose a patient from the HPN population at random. The adversary would not know whether that individual was in the HHP dataset, and hence this would introduce some uncertainty. If the individual was in the dataset then we computed the appropriate *p_ih_* value for each quasi-identifier for that individual, and then selected the items of background knowledge about the individual. We then attempted to match the background knowledge with all of the patients in the competition dataset. The simulation was run 10,000 times, and the average match success rate gave us an estimate of the probability that an HPN patient could be correctly re-identified from the competition dataset under the assumptions that we made.

The purpose of the simulated attack was to mimic what an adversary would do. We assumed that the adversary had background information about Alice. Alice may be the adversary’s neighbor or a famous person. She could also be someone the adversary selected at random from all HPN members.

The simulation dataset had two levels. Level 1 was the basic patient demographics as in Table 2. At level 2 were the quasi-identifiers in each claim, and a patient may have had a large number of claims. The level 2 data had some quasi-identifiers as shown in Table 3.

We also needed to create two versions of the de-identified dataset. Version D1 of the dataset had all of the claims for each patient. Version D2 of the dataset was the one with truncated claims. It is version D2 of the dataset that was released for the competition, but we needed D1 for the simulation. The level of generalization in the two datasets was exactly the same, the only difference being in the truncation of claims.

The following process was repeated 10,000 times:

- We drew a sample from a binomial distribution with a probability of α. This reflects the probability that an individual that the adversary knew about was in the dataset. If the value drawn was 1, then we could continue; otherwise, we would go to the next iteration (and the current iteration was considered a failed match).
- Then we chose a target individual from the D1 dataset at random.
- We chose at random *p_ih_* values for each level 2 quasi-identifier from the D1 dataset for that target individual. These values and the level 1 quasi-identifiers were the background information that the adversary would have.
- We matched that background information to the records in D2. This produced a matching equivalence class.
- One of the records was selected in the matching equivalence class at random.
- If the selected record was the correct patient then that was a successful match; otherwise, it was considered a failure.

Across the 10,000 iterations we computed the proportion of times that a correct match was found. This was the re-identification probability for the dataset taking into account the uncertainty due to the fact that we had a sample and due to the adversary not knowing which claims were truncated.

#### Attack 2

To compute marketer risk [29] for attack 2 we assumed that the voter registration list captured the full population, an assumption used in previous research as well [35]. We first needed to calculate the size of the equivalence classes in the population of California in the counties serviced by HPN. In this case, the equivalence classes were defined by the number of people born of each sex, in one of the counties of interest, and of each age. To compute this size, we took age and sex values from the 2000 census (the 2010 census data were not available at the time we performed this analysis), which were available at the county level for 5-year intervals (top-coded to 90+) on the American FactFinder website from the Census Bureau. This produced 20 equivalence classes per county. We derive in Multimedia Appendix 1 a closed-form equation for the expected number of records that would be correctly matched when a sample of a given size is drawn from a population. The derivation allowed us to compute expected marketer risk without having to perform an actual matching experiment or a Monte Carlo simulation.

#### Attack 3

We estimated the proportion of HHP records that could be correctly matched with the SID on the quasi-identifiers using the closed-form marketer risk calculation described in Multimedia Appendix 1. Here we assumed that the HHP would be a sample from the SID. The marketer risk calculations were performed for different combinations of quasi-identifiers. We assumed that the HHP dataset had all of the visits in the SID, and therefore all of the visits could be used for matching. We purchased the SID for the state of California from AHRQ for the 3 years covered by the HHP dataset to perform this analysis.

### Sensitivity Analysis

We also analyzed sensitivity for the assumptions we made under attack 1. We explored three relaxations to the assumptions:

- The maximum power of the adversary, *p_m_*, was higher than our assumed value of 5. We set the power to 10 and then to 15. With 6 quasi-identifiers, this would mean that the adversary knew up to 60 to 90 pieces of information about the patients they were attempting to re-identify.
- For 1 claim the adversary knew all of the quasi-identifiers for that claim. For example, say that we had only 2 quasi-identifiers, LOS and Diagnosis. Then we would assume that the adversary knew the LOS and Diagnosis values for the same claim. This relaxes the inexact knowledge assumption.
- The adversary knew the order of 1 pair of quasi-identifier values. For example, the adversary would know that diagnosis A preceded diagnosis B. This would apply only in cases where the power for the quasi-identifier was greater than 1. We would apply this for a pair of claims for each quasi-identifier. This relaxes the inexact order assumption.

With these three types of sensitivity analyses we believed we covered plausible scenarios in which the adversary would have extensive knowledge about the individuals in the competition dataset.

## Results

The final claims dataset consisted of information from 113,000 patients, with 2,668,990 claims. The median number of claims per person was 11 and the maximum 136. Only 9556 patients had some of their claims truncated during the de-identification.

Making the conservative assumption that 0.8% of the individuals with a probability of re-identification higher than our threshold of .05 would have a probability of re-identification of 1, we would expect at most 5.8% of the patients to be re-identified based on our de-identification parameters.

After applying the LOLA algorithm to determine the optimal generalizations, we obtained the final results presented in Table 5. With these generalizations, 0.84% of the patients could be correctly re-identified using our simulated attack 1.

The risk calculation for attack 2 was that an expected proportion of 0.0005% of the HHP dataset could be correctly re-identified by matching with the appropriate counties in the California voter registration list. Furthermore, there are restrictions on the use of the California voter registration list that would prohibit such re-identification attempts [35]. Therefore, attack 2 was deemed to be very low risk.

The results for attack 3 are shown in Table 6 for various combinations of quasi-identifiers by year and across all years. As shown, the match success proportion was quite low, making the risk of gaining correct additional information about the HHP patients acceptable given our thresholds.

Table 7 shows the results of the simulation attack to evaluate sensitivity to violations of our assumptions for attack 1. The re-identification probability was not affected much by the increase in the power of the adversary. A primary reason was that many patients had 5 claims or fewer. Therefore, increasing the power did not necessarily mean that the adversary would have more background information about them. If we assume that the adversary would know which pieces of information were in the same claim, this would increase the risk, but even at a power of 15 the probability was below what would be considered acceptable.

**Table 5:** Final generalizations in the dataset.

Quasi-identifier	Generalization
Age	10-year interval; 80+
Sex	No change
DaysInHospital Y2	Days to 2 weeks; >2 weeks in year 2
DaysInHospital Y3	Days to 2 weeks; >2 weeks in year 3
Specialty	Grouped specialty (see Multimedia Appendix 1)
PlaceOfService	Grouped place of service (see Multimedia Appendix 1)
CPTCode^a^	Grouped CPT code (see Multimedia Appendix 1)
LOS^b^	Days up to 6 days; (1–2] weeks; (2–4] weeks; (4–8] weeks; (8–12 weeks]; (12–26] weeks; 26+ weeks
DSFC^c^	4 weeks
Diagnosis	Primary condition group (see Multimedia Appendix 1)

^a^ Current Procedural Terminology.

^b^ Length of stay in hospital.

^c^ Days since first claim.

**Table 6:** Estimated proportion of all records in the Heritage Health Prize dataset that would be correctly matched against the State Inpatient Database.

Age	LOS^a^	Sex	Number of visits	PCG^b^	CPT^c^	Year 1	Year 2	Year 3	All years
X	X	X	X			0.001612	0.001478	0.001515	0.005141
X	X	X			X	0.007105	0.005684	0.005965	0.009735
X	X	X		X		0.013334	0.010156	0.010928	0.013579
X	X	X		X	X	0.017272	0.012702	0.013797	0.015991

^a^ Length of stay in hospital.

^b^ Primary Condition Group.

^c^ Current Procedural Terminology.

**Table 7:** Percentage of total records correctly matched under simulated attack with different assumptions about the number of claims (power).

	Power of adversary		
Assumption	5	10	15
Original adversary assumptions	0.84%	0.94%	1.17%
Multiple quasi-identifiers in the same claim	3.67%	3.72%	3.87%
Ordered claims	0.96%	1.0%	1.2%

## Discussion

### Summary

The detailed re-identification risk assessment on the HHP dataset allowed the disclosure of comprehensive longitudinal claims information on a large number of individuals while being able to make strong statements about the ability to re-identify these individuals. The de-identification we performed ensured that the risk was acceptable under different types of attacks, even to the extent that we allowed for some of our initial assumptions to be incorrect. In particular, we were able to ensure that the risk exposure was at or below the current risk exposure under the HIPAA Safe Harbor de-identification standard.

Ensuring the utility of the dataset is an important requirement in any de-identification effort. If no team is able to meet the prediction performance threshold to win the grand prize, then this may be because the threshold was too ambitious or because the de-identification itself made achieving that threshold difficult. An evaluation of the accuracy of the models before and after de-identification would be a useful exercise to help inform future competitions and fine-tune de-identification methods.

As our literature review in Multimedia Appendix 1 illustrates, existing de-identification methods for longitudinal data would not have created a dataset suitable for this competition. In that regard, the approach presented here is one of the few available for creating public health datasets.

### Limitations

Alternative ways for grouping the diagnosis and procedure codes could have been used. For example, we could have clustered the codes based on the average number of days of hospitalization. This would potentially have retained some important relationships in the data. Furthermore, it would ideally be necessary to perform this clustering using all of the quasi-identifiers to ensure that the multivariate relationships are retained. The practical challenge with such an approach was that many patients had zero days in hospital (for example, they were outpatients). This would then have resulted in coarser groupings than those we included with our analysis. Appropriate grouping of such nominal variables is an important area of future research to address constraints imposed by real datasets.

We did not consider the real possibility that there were errors in the background knowledge of the adversary. If errors exist then the match percentages would be lower than those we presented in our results.

Our analysis did not address risks from attribute disclosure. As noted earlier, there are no known attribute disclosure attacks on health data, and the HIPAA Privacy Rule does not require the management of attribute disclosure. This makes it difficult to determine what acceptable risk standards for attribute disclosure might be. Nevertheless, it would be appropriate to develop acceptable standards for managing attribute disclosure for future data releases.

## Multimedia Appendix 1

Technical description of the de-identification methods.

## Abbreviations

AHRQ: Agency for Healthcare Research and Quality
CPT: Current Procedural Terminology
HHP: Heritage Health Prize
HIPAA: Health Insurance Portability and Accountability Act
HPN: Heritage Provider Network
ICD-9-CM: International Classification of Diseases, 9th revision, clinical modification
LOLA: longitudinal optimal lattice anonymization
LOS: length of stay
OLA: optimal lattice anonymization
PCG: primary condition group
SID: State Inpatient Database

## References

[1] Fienberg SE (1994). Sharing statistical data in the biomedical and health sciences: ethical, institutional, legal, and professional dimensions. Annu Rev Public Health.

[2] Sztompka P (2007). Trust in science: Robert K Merton's inspirations. J Classical Sociol.

[3] Fienberg SE, Martin ME, Straf ML (1985). Sharing Research Data.

[4] Sieber JE (1988). Data sharing: defining problems and seeking solutions. Law Hum Behav.

[5] Hedrick T (1988). Justifications for the sharing of social science data. Law Hum Behav.

[6] Stanley B, Stanley M (1988). Data sharing: the primary researcher's perspective. Law Hum Behav.

[7] Kirwan JR (1997). Making original data from clinical studies available for alternative analysis. J Rheumatol.

[8] Hrynaszkiewicz I, Altman DG (2009). Towards agreement on best practice for publishing raw clinical trial data. Trials.

[9] Piwowar HA, Day RS, Fridsma DB (2007). Sharing detailed research data is associated with increased citation rate. PLoS One.

[10] Tananbaum G (2008). Adventures in open data. Learn Publ.

[11] Murray-Rust P (2008). Open data in science. Ser Rev.

[12] Perry CM (2008). Archiving of publicly funded research data: a survey of Canadian researchers. Gov Inf Q.

[13] Duncan GT, Jabine TB, de Wolf VA (1993). Private Lives and Public Policies: Confidentiality and Accessibility of Government Statistics.

[14] (2011). Heritage Provider Network Health Prize.

[15] (2010). Kaggle Inc.

[16] (2009). Predictive Modeling Group, IBM Research Center.

[17] Phung H (2012). Practice Fusion.

[18] Practice Fusion (2010). Microsoft.

[19] El Emam K, Paton D, Dankar F, Koru G (2011). De-identifying a public use microdata file from the Canadian national discharge abstract database. BMC Med Inform Decis Mak.

[20] Obama B (2009). The White House.

[21] El Emam K, Jonker E, Arbuckle L, Malin B (2011). A systematic review of re-identification attacks on health data. PLoS One.

[22] Association AM (2006). Cpt 2006 Professional (Cpt / Current Procedural Terminology (Professional Edition)).

[23] World Health Organization (2011). Centers for Disease Control and Prevention.

[24] International Organization for Standardization (2009). ISO/TS 25237:2008: Health Informatics: Pseudonymization.

[25] El Emam K (2008). Heuristics for de-identifying health data. IEEE Secur Priv.

[26] Tamersoy A, Loukides G, Denny J, Malin B (2010). Anonymization of administrative billing codes with repeated diagnosis through censoring. AMIA Annu Symp Proc.

[27] Eguale T, Bartlett G, Tamblyn R (2005). Rare visible disorders/diseases as individually identifiable health information. AMIA Annu Symp Proc.

[28] El Emam K, Dankar FK (2008). Protecting privacy using k-anonymity. J Am Med Inform Assoc.

[29] Dankar F, El Emam K (2010). A method for evaluating marketer re-identification risk. Proceedings of the 2010 EDBT/ICDT Workshops.

[30] Xu Y, Fung B, Wang K, Fu A, Pei J (2008). Publishing sensitive transactions for itemset utility. Proceedings of the Eighth IEEE International Conference on Data Mining.

[31] Xu Y, Wang K, Fu A, Yu P (2008). Anonymizing transaction databases for publication. Proceedings of the 14th ACM SIGKDD international conference on Knowledge discovery and data mining.

[32] Terrovitis M, Mamoulis N, Kalnis P (2008). Privacy-preserving anonymization of set-valued data. Proceedings VLDB Endowment.

[33] Sweeney L (2001). Computational Disclosure Control: A Primer on Data Privacy Protection dissertation.

[34] El Emam K, Dankar F, Vaillancourt R, Roffey T, Lysyk M (2009). Evaluating patient re-identification risk from hospital prescription records. Can J Hosp Pharm.

[35] Benitez K, Malin B (2010). Evaluating re-identification risks with respect to the HIPAA privacy rule. J Am Med Inform Assoc.

[36] Consumer-Purchaser Disclosure Project (2004). The State Experience in Health Quality Data collection.

[37] El Emam K, Mercer J, Moreau K, Grava-Gubins I, Buckeridge D, Jonker E (2011). Physician privacy concerns when disclosing patient data for public health purposes during a pandemic influenza outbreak. BMC Public Health.

[38] El Emam K, Dankar FK, Issa R, Jonker E, Amyot D, Cogo E, Corriveau JP, Walker M, Chowdhury S, Vaillancourt R, Roffey T, Bottomley J (2009). A globally optimal k-anonymity method for the de-identification of health data. J Am Med Inform Assoc.

[39] Samarati P, Sweeney L (1998). Protecting privacy when disclosing information: k-anonymity and its enforcement through generalisation and suppression. Technical Report SRI-CSL-98-04.

[40] Samarati P (2001). Protecting respondents' identities in microdata release. IEEE Trans Knowl Data Eng.

[41] Sweeney L (2002). k-Anonymity: a model for protecting privacy. Int J Uncertain Fuzziness Knowl Based Syst.

[42] Ciriani V, De Capitani di Vimercati S Foresti S, Samarati P (2007). k-Anonymity. Yu T, Jajodia S, editors. Secure Data Management in Decentralized Systems.

[43] Department of Health and Human Services (2000). Federal Register.

[44] Department of Health and Human Services (2000). Federal Register.

[45] Cohn SP (2007). Report to the Secretary of the US Department of Health and Human Services on Enhanced Protections for Uses of Health Data: A Stewardship Framework for “Secondary Uses” of Electronically Collected and Transmitted Health Data.

[46] Sweeney L (2010). Data sharing under HIPAA: 12 years later.

[47] Cancer Care Ontario (2005). Data Use & Disclosure Policy.

[48] Health Quality Council (2004). Security and Confidentiality Policies and Procedures.

[49] Health Quality Council (2004). Privacy Code.

[50] Manitoba Center for Health Policy (2002). Privacy Code.

[51] Subcommittee on Disclosure Limitation Methodology, Federal Committee on Statistical Methodology (1994). Statistical Policy Working Paper 22: Report on Statistical Disclosure Limitation Methodology.

[52] Statistics Canada (2006). Therapeutic Abortion Survey.

[53] Information and Privacy Commissioner of British Columbia (1998). Order No 261-1998: Inquiry re: A Media Request for Access to Records Regarding the Administration of the Medication Ritalin by School District Staff to Elementary School Students.

[54] Information and Privacy Commissioner of Ontario (1994). Order P-644, Appeal P-9300524, Ministry of Health.

[55] Alexander LA, Jabine TB (1978). Access to social security microdata files for research and statistical purposes. Soc Secur Bull.

[56] Ministry of Health and Long-Term Care (1984). Corporate Policy 3-1-21.

[57] de Waal A, Willenborg L (1996). A view on statistical disclosure control for microdata. Surv Methodol.

[58] Office of the Privacy Commissioner of Quebec (CAI) (1997). Chenard v Ministere de l'agriculture, des pecheries et de l'alimentation (141).

[59] Seastrom MM (2003). NCES Statistical Standards.

[60] Arkansas HIV/AIDS Surveillance Section (2010). Arkansas HIV/AIDS Data Release Policy.

[61] Centers for Medicare & Medicaid Services (2011). BSA Inpatient Claims PUF.

[62] Escobar GJ, Greene JD, Scheirer P, Gardner MN, Draper D, Kipnis P (2008). Risk-adjusting hospital inpatient mortality using automated inpatient, outpatient, and laboratory databases. Med Care.

[63] Charlson ME, Pompei P, Ales KL, MacKenzie CR (1987). A new method of classifying prognostic comorbidity in longitudinal studies: development and validation. J Chronic Dis.

[64] Quan H, Sundararajan V, Halfon P, Fong A, Burnand B, Luthi JC, Saunders LD, Beck CA, Feasby TE, Ghali WA (2005). Coding algorithms for defining comorbidities in ICD-9-CM and ICD-10 administrative data. Med Care.

[65] Liu J, Wang K (2010). Anonymizing transaction data by integrating suppression and generalization. Advances in Knowledge Discovery and Data Mining, Lecture Notes in Computer Science.

[66] He Y, Naughton J (2009). Anonymization of set-valued data via top-down, local generalization. Proceedings VLDB Endowment.

[67] Davison A, Hinkley D (1997). Bootstrap methods and their application.

