# Supplementary material for: De-identification Methods for Open Health Data: The Case of the Heritage Health Prize Claims Dataset
Source: J Med Internet Res. 2012 Feb 27;14(1):e33. doi: 10.2196/jmir.2001 (PMC3374547; doi:10.2196/jmir.2001)
Supplement: Supplementary file 1 [file jmir_v14i1e33_app1.pdf]

Multimedia Appendix

This appendix provides the detailed analysis that was performed for the de-identification of the Heritage Health Prize data set.

1 Truncation of Claims

The number of claims per patient were truncated at the 95th percentile. All claims for a patient were ordered by a score, and then claims with the highest score were suppressed to meet the 95th percentile threshold. Our approach to computing the score for each claim was based on support, where the higher the score, the higher the likelihood that the claim would be truncated. Support was denoted by the function $\sup(X)$, and was computed as the number of other patients with the value X. A score was defined based on the support values for the quasi-identifiers in a claim.

Let us say that a claim had $a_{ir1} \ldots a_{irh}$ quasi-identifiers, where i was an index for the patient, r an index for the claim, and h an index for quasi-identifiers. We wished to assign a higher score to claims with quasi-identifiers that had low support. If we had N patients in the data set, then we could compute the score as $s_{ir} = 1 - \frac{\min_h(\sup(a_{irh}))}{N}$. This score gave the quasi-identifier with the lowest support the most weight in deciding the overall score for a claim. The reasoning for this was that if any claim had a quasi-identifier that was quite rare in the data set (among patients) then its score would be quite high.

2 Removal of High Risk Patients

The following patient groups were removed from the data set during the pre-processing step:

- Patients with diagnoses indicating Human Immunodeficiency Virus (HIV), abortion, abuse, psychosexual disorders, mental retardation, or plastic surgery:
    - Human immunodeficiency virus (HIV) infection (ICD-9 codes 042–044)
    - Other pregnancy with abortive outcome (ICD-9 codes 634–639)
    - Ectopic and molar pregnancy (ICD-9 codes 630–633)
    - Physical, psychological, and sexual abuse (including self inflicted) or assault (ICD-9 codes 995.5, 995.81, 995.82, 995.83, E95, E96, V15.4)
    - Substance abuse or dependence (ICD-9 codes 291, 292, 303, 304, 305)
    - Psychosexual disorders (ICD-9 codes 302) or mental retardation (ICD-9 codes 317-319)
    - Plastic surgery for unacceptable cosmetic appearance (ICD-9 code V50.1) or aftercare involving the use of plastic surgery (ICD-9 code V51).

  - Patients having undergone procedures indicating intersex surgery (CPT codes starting with 5597, or 5598).
  - Patients who had abortions (CPT codes starting with 59100, 59812, 59820, 59821, 59830, 59840, 59841, 59850-59852, 59855-59857)
  - Patients with alcohol/drug abuse or dependence (CPT chapter 897).
- Patients with diagnoses indicating a rare and visible disease [1].
- Patients with a claim where the “place of service” is Intermediate Care—Mentally Retarded, Residential or Nonresidential Substance Abuse Treatment Facility (codes 55-57).

The following claims were removed because they would indicate an important event in a patient’s life, making them more easily identifiable, or were not considered relevant:

- Deleted claims for newborns (up to and including 28 days after birth).
- Claims where the CPT code is not a medical procedure (all codes with a letter prefix, known as level II codes, and three digit revenue or chapter codes).

When a high-risk patient or claim was deleted from the data set, all claims with the same MemberID were removed from the data. The concern behind this decision was the possibilty that information in other claims for the same patient can be used to infer the deleted information. For example, if only a claim with a direct diagnosis of HIV is deleted, but then other claims with diagnoses of infections that are very strongly related would allow an adversary to infer that the patient did have HIV.

3 Differences Between Excluded and All Patients

In this section we describe the difference between the high risk patients that were removed during the previous step and the remaining patients. In Table 1 we see that the distribution on gender is quite similar.

On the other hand, there were differences on some of the variables. We found that the excluded patients tended to have a longer interval between claims (Table 2), be older (Table 3), and to require longer stays in hospital (Table 4). This is not surprising in that the diagnoses and procedures that were excluded are most likely to occur with an older population. The median number of claims per patient that was excluded was 34 compared to 11 for the remaining patients. This indicates that the excluded patients tended to have more procedures performed compared to the general patient population, and is testimony to the fact that their conditions tend to be serious and often chronic ones.

**Sex**	**Excluded Patients (%)**	**Remaining Patients (%)**
**Male**	48.71	46.41
**Female**	51.29	53.59

Table 1: Comparison of excluded patients with the remaining patients in terms of sex.

DSFC	Excluded Patients (%)	Remaining Patients (%)
**0-1 month**	19.38	25.89
**1-2 months**	9.57	9.52
**2-3 months**	8.85	8.72
**3-4 months**	8.58	8.24
**4-5 months**	7.9	7.37
**5-6 months**	8.12	7.46
**6-7 months**	7.64	6.99
**7-8 months**	7.51	6.8
**8-9 months**	7.51	6.61
**9-10 months**	6.67	5.63
**10-11 months**	5.57	4.58
**11-12 months**	2.72	2.0

Table 2: Comparison of excluded patients with the remaining patients in terms of DSFC. A two-sample Kolmogorov-Smirnov test found strong evidence ($p<0.0001$) that the distribution of DSFC between the two groups was different.

Age	Excluded Patients (%)	Remaining Patients (%)
**0-9**	3.11	10.32
**10-19**	5.16	10.83
**20-29**	5.92	8.63
**30-39**	9.77	12.19
**40-49**	16.10	15.27
**50-59**	15.55	12.62
**60-69**	17.99	11.66
**70-79**	18.84	12.00
**80+**	7.57	6.48

Table 3: Comparison of excluded patients with the remaining patients in terms of age. A two-sample Kolmogorov-Smirnov test found strong evidence ($p<0.0001$) that the distribution of age between the two groups to be different.

LOS	Excluded Patients (%)	Remaining Patients (%)
**1 day**	48.77	58.2
**2 days**	10.3	9.84
**3 days**	7.29	6.47
**4 days**	5.09	4.07
**5 days**	3.08	2.58
**6 days**	2.14	1.42
**1-2 weeks**	0.62	0.27
**2-4 weeks**	8.27	6.27
**4-8 weeks**	7.26	5.54
**8-12 weeks**	5.92	4.54
**12-26 weeks**	0.52	0.43
**26+ weeks**	0.75	0.37

Table 4: Comparison of excluded patients with the remaining patients in terms of LOS. A two-sample Kolmogorov-Smirnov test found strong evidence ($p<0.0001$) that the distribution of LOS between the two groups to be different.

4 Computing the Adversary Power

In this section we describe how we computed the power of the adversary by considering the number of claims that a patient has and the diversity or variability in their quasi-identifier values.

We made two assumptions about the knowledge of the adversary: (a) the adversary would not know which values on the quasi-identifiers were in the same claim (the “inexact knowledge” assumption), and (b) the adversary would not know the exact order of the claims (the “inexact order” assumption) beyond what is revealed through the DSFC quasi-identifier, which is consistent with other models of transactional data in the disclosure control literature [2-6]. However, we did test the sensitivity of our results to these assumptions in our empirical evaluation.

For the first assumption, for example, the adversary could know that a patient had a heart attack and stayed for a week at the hospital during the period covered by the data, but would not know that these two values pertained to the same episode of care. For instance, a patient could have had the following series of primary condition groups (a generalization of the diagnosis code): <AMI, AMI, UTI, RENAL3> and a series of LOS values <2,4,1,7>, but the values in the two quasi-identifier sequences were not ordered the same way. The adversary could know that there were two AMI diagnoses but not know that the LOS was 7 days for one of them.

For the second assumption, we assumed that the adversary would not necessarily know the exact order of the quasi-identifier values. For example, if an AMI and UTI diagnoses occurred within the same day, say, the adversary would not know that the UTI occurred before the heart attack. These two diagnoses would have two separate claims which shared the same date.

For each individual, we defined their variability π_{ih}, which would start from zero, indicating no variability in the quasi-identifier values for that patient. This characterized how often the values in their set of quasi-identifier values would vary. For example, a patient with a chronic disease, such as kidney disease, who made many dialysis visits, would have low variability in their diagnosis codes across many claims. On the other hand, a patient with multiple acute incidents would have high variability in their diagnosis codes since one would expect that they would be unrelated to each other.

A patient with low variability would be easier to re-identify because an adversary would need to know little about them to re-identify them. An adversary who knew little would be able to predict information in the rest of the claims because there was little variability. Consequently, the adversary would be likely to have background information about many of the quasi-identifier values, which would make p value higher.

For a patient who had high variability, every additional new piece of information about the patient would be so different from previous information, the adversary could not use existing knowledge to figure out other information. This means that the adversary would be likely to have less background information about a patient and hence p would be lower.

We also defined η_i as the number of claims that a patient had. The number of claims was independent of the quasi-identifier as that number was constant across all quasi-identifiers. The more claims that a patient had, the more information that was available to be used for re-identification. Therefore, it would be easier for an adversary to have more background information about patients who had many claims.

		π_{ih}	
		Low	High
η_i	Few	(1) $p_{ih} = 5$	(3) $p_{ih} = 3$
	Many	(2) $p_{ih} = 7$	(4) $p_{ih} = 5$

Figure 1: The four quadrants showing different adversary power levels.

We could then define conceptually four quadrants of patients as in Figure 1. For patients in quadrant (3) little information would be available to an adversary because there were few claims and there was so much diversity in the patient's information (i.e., it would be more difficult to use known information to predict additional information). Therefore, we assigned them a low p value. On the other hand, for patients in quadrant (2) it would be easier to get more background information about them because they had so many similar claims that knowing a little the adversary could predict others. Therefore the power of the adversary would be much higher. The other quadrants were in between.

Let the p value for quadrant (1) be denoted by $p(1)$. The basic relationships among the p values are $p(1) \leq p(2)$, $p(3) \leq p(4)$, $p(1) \geq p(3)$, and $p(2) \geq p(4)$. Therefore, we expected a monotonic relationship between η_i / π_{ih} and the p value. If we strengthen that monotonic assumption and say that the relationship is linear then the value of p can be computed as follows:

$$p_{ij} = \left\lceil \frac{p_m - 1}{\max_i \left(\frac{\eta_i}{\pi_{ih}} \right)} \left(\frac{\eta_i}{\pi_{ih}} \right) + 1 \right\rceil \qquad \text{..............................(1)}$$

where p_m is the maximum value of p that we were willing to assume.

We set the value of p_m at 5. While there are no precedents for this number, it represented a significant amount of background information about the patients: for 6

quasi-identifiers in each claim, this would mean that the adversary could have up to 30 pieces of information about the patient to use for re-identification, plus the 4 quasi-identifiers in the patients' table. This was a significant amount of background information and therefore represented quite a knowledgeable adversary. Because many patients had more claims than 5, it was also assumed that all claims were equally probable to be within the background knowledge of the adversary. As part of our empirical evaluation we evaluated the sensitivity of our results to the use of $p_m = 5$.

In some cases where there was absolutely no variability in the data, the π_{ih} would be zero. This means that the $\left(\frac{\eta_i}{\pi_{ih}}\right)$ value could not be computed in the equation above. To deal with such cases we computed $\max_i\left(\frac{\eta_i}{\pi_{ih}}\right)$ only for $\pi_{ih} > 0$. For any i, j if $\pi_{ih} = 0$ then the $\left(\frac{\eta_i}{\pi_{ih}}\right)$ term in equation (1) was set equal to $\max_i\left(\frac{\eta_i}{\pi_{ih}}\right)$ where $\pi_{ih} > 0$. In such a case if there was no diversity in the data then the maximum value of p was always selected.

If there were patients with an extreme number of claims, they would skew the calculations of p_{ih} lower. Therefore we capped the value of η_i at the mean and two standard deviations. This meant that if a member had more claims than that, these additional claims were not considered to provide the adversary with additional information for a re-identification attack. In practice this cutoff was still quite high, but did prevent extreme skewness in the distribution of p_{ih}.

4.1 Computation of Variability

Although the Shannon index is commonly used for estimating variability, it is sensitive to sample size and difficult to interpret [7]. Instead, we estimated variability using the more robust Simpson index [8].

Let X be a categorical variable with k categories, n_k be the frequency of occurrence of category k, and N be the frequency of occurrence across all categories (i.e., $N = \sum n_k$). For a finite population, the Simpson index is calculated as $D = \sum \frac{n_k(n_k - 1)}{N(N-1)}$, and represents the probability of two randomly selected occurrences being in the same category.

For example, if X_h is primary condition groups (quasi-identifier h) then $k = 1, \ldots, 45$. Assume patient i has a vector of primary conditions $X_{ih} = (\text{AMI, AMI, AMI, UTI, RENAL3, RENAL3})$, then $n_1 = 3$, $n_2 = 1$, $n_3 = 2$, and $N = 6$. Therefore, the Simpson index would be $D = \frac{3(2)}{6(5)} + \frac{3(2)}{6(5)} + \frac{3(2)}{6(5)} \approx 0.27$

Note, however, the degenerate case when $N = 1$, resulting in a divide by zero. In this case we let the Simpson index $D = 1$.

4.2 Calculating diversity from the Simpson index

To determine diversity from the Simpson index, one can either take the complement $(1-D)$, or the reciprocal $(1/D)$. Although the reciprocal $(1/D)$ is commonly used, it can have variance problems. Some therefore recommend using $\ln(D)$. Still, others argue for using $(1-D)$, which is easily interpretable as it is a probability. We therefore chose to use this latter measure of diversity.

4.3 Correlation Among Diversity Values

The correlation matrix of the diversity values among the quasi-identifiers is shown in Table 5. This shows that the relationship among variables in terms of their diversity varies considerably, ranging from negative moderate, to close to zero, to moderate positive. While the signs and magnitudes of these correlations have face validity, they also re-enforce the need to treat the diversity of each quasi-identifier separately in computing the power of the adversary, rather than attempting to compute a single diversity value across all claims.

	**Year**	**Specialty**	**PlaceOfService**	**LOS**	**DSFC**	**Diagnosis (PCG)**	**CPTCode (generalized)**
**Year**	1	-0.021	-0.057	-0.232	0.103	0.147	-0.036
**Specialty**		1	0.461	0.084	0.063	0.287	0.413
**PlaceOfService**			1	0.275	-0.085	0.140	0.451
**LOS**				1	-0.44	-0.175	0.306
**DSFC**					1	0.215	-0.064
**Diagnosis (PCG)**						1	0.063
**CPTCode (generalized)**							1

Table 5: Correlation matrix among the diversity values for the quasi-identifiers.

5 Node Computation

Below we describe the precise steps in computing the re-identificatiion risk for each node in the lattice.

We used i to index patients and h to index quasi-identifiers in a claim. The following are the calculation steps followed within a node. The objective of the processing within the node was to determine if this was a candidate solution node or not:

1. The data is generalized according to the specifications for the node. Let this be data set D.
2. For each quasi-identifier at Level 2 and each patient, compute the p_{ij} value.

   We compute the value of p before truncation because we want to reflect how much information the adversary would have without consideration of what we did to the data. An adversary may have extensive background information about a patient, but this would be useless to him/her if those claims are truncated, and we wanted to reflect that when computing the risk for the node.
3. Based on the generalization for the number of claims, select the patients who need to have their claims truncated and apply the truncation. Let this be data set D'
   Truncation means the removal of those claims from further consideration in this node. Because the claims are removed they cannot be treated as quasi-identifiers useful for re-identification.
4. Repeat 1,000 times (or when the stopping criterion is met)
   a. Draw 10,000 patients with replacement
   b. For each patient i
      i. For each quasi-identifier h
         1. Sample p_{ih} values from data set D
         2. If any of the sampled p_{ih} values are not in the data set D' for that patient then this patient is considered low risk, set $i = i + 1$, and go to 4(a)
            If the adversary knows background knowledge that is not in the data set because it was truncated, then it is not possible to have a successful match by definition.
      ii. Compute the size of the equivalence class for that patient on all of the sampled quasi-identifier values
      iii. If the equivalence class is smaller than the threshold then flag this patient as high risk, set $i = i + 1$, and go to 4(a)
   c. Compute the proportion of patients that are high risk
5. If the mean proportion of high risk patients (i.e., flagged for suppression) $> MaxSup$ then this node is not a candidate and exit the node calculation, otherwise this is a candidate node

6 Derivation of Sample Marketer Risk

We let J be the set of equivalence classes in the population data set, with N records. The size of each equivalence class was given by F_j where $j \in J$. A sample was drawn from the population. The set of equivalence classes in the sample was denoted by S,

such that $S \subseteq J$. The sample size was given by n, and the size of an equivalence class in the sample was given by f_j where $j \in S$.

Marketer risk was given by $\theta = \frac{1}{n}\sum_{j \in S}\frac{f_j}{F_j}$ [9]. We had $f_j = \alpha F_j$. Therefore, we ended up with $\theta = \frac{1}{n}\sum \alpha = \frac{|S|\alpha}{n}$, where $|S|$ was the number of equivalence classes in the sample. To determine the value of θ we needed to compute the value of $|S|$.

Assuming we would randomly draw n records from the population. We let U_j be a random variable associated with each f_j such that

$$U_j = 0 \; if \; f_j = 0$$

$$U_j = 1 \; if \; f_j > 0$$

then:

$$\begin{aligned} E(U_j) &= 0 \times P(f_j = 0) + 1 \times P(f_j > 0) \\ &= P(f_j > 0) = 1 - P(f_j = 0) \\ &= 1 - \frac{\binom{N - F_j}{n}}{\binom{N}{n}} \\ &= 1 - \frac{(N - F_j)!(N-n)!}{(N-n-F_j)!N!} \end{aligned}$$

We let $U = \sum_{j \in J} U_j$, which represented the non-zero equivalence classes in the sample. Then:

$$\begin{aligned} E(U) &= E\left(\sum_{j \in J} U_j\right) = \sum_{j \in J} E(U_j) \\ &= \sum_{j \in J}\left(1 - \frac{(N - F_j)!(N-n)!}{(N-n-F_j)!N!}\right) \end{aligned}$$

Putting this in the equation for θ, we had an approximation:

$$\hat{\theta} = \frac{1}{N}\sum_{j\in J}\left(1-\frac{(N-F_j)!(N-n)!}{(N-n-F_j)!N!}\right)$$

The above equation could be simplified to:

$$\begin{aligned}\hat{\theta} &= \frac{1}{N}\sum_{j\in J}\left(1-\frac{(N-F_j)!(N-n)!}{(N-n-F_j)!N!}\right)\\ &= \frac{1}{N}\sum_{j\in J}\left(1-\frac{\prod_{i=0}^{F_j-1}(N-n-i)}{\prod_{i=0}^{F_j-1}(N-i)}\right) \qquad \text{.. (2)}\\ &= \frac{1}{N}\sum_{j\in J}\left(1-\prod_{i=0}^{F_j-1}\left(1-\frac{n}{N-i}\right)\right)\end{aligned}$$

Based on that calculation, the proportion of HHP patients that could be correctly matched to a voter list, on average, would be calculated as $\hat{\theta}$ in equation (2).

7 Grouping ICD-9 Codes into Primary Condition Groups

The following is a summary of how ICD-9 codes were grouped into larger sets for the primary condition groups [10]:

PRIMARY CONDITION GROUP	DESCRIPTION & INCLUDED ICD-9 CODES
**ACUTE MYOCARDIAL INFARCTION**<br><br>AMI	Myocardial infarction.<br><br>410-414
**ACUTE RENAL FAILURE**<br><br>RENAL1	Acute renal failure, nephrotic syndrome, and related conditions.<br><br>580, 581, 584
**ACUTE RESPIRATORY**<br><br>RESPR4	Acute respiratory infections and miscellaneous respiratory diseases.<br><br>460-478, 786
**ALL OTHER INFECTIONS**<br><br>INFEC4	All other infections, joint infections and muscle infections, with the exception of hepatitis; unspecified fever.<br><br>001-139, multiple others, incl. joint infections & muscle infections (711 & 728); 780.6 (fever)
**ALL OTHER TRAUMA**	Traumatic injuries not included elsewhere, including head

TRAUMA	injuries without intracranial or subdural bleeds.<br><br>800-804, 840-848, 850-854, 860-904, most of 905-959
**APPENDICITIS**<br><br>APPCHOL	Appendicitis, hernias, cholecystitis, and cholangitis.<br><br>540-543, 550-553, 574-576
**ARTHROPATHIES**<br><br>ARTHSPIN	Arthropathies and spine disorders (but no infections or autoimmune conditions).<br><br>712, 715-729, most 731-739 (except for 733.1xx, pathologic fracture)
**ATHEROSCLEROSIS AND PERIPHERAL VASCULAR DISEASE**<br><br>HEART4	Atherosclerosis (including that affecting precerebral arteries) and other forms of peripheral vascular disease.<br><br>429.2, 433, 440-459
**CANCER A**<br><br>CANCRA	Malignant neoplasms of respiratory tract and intrathoracic organs; leukemias, non-Hodgkin's lymphomas, and other histiocytic malignancies.<br><br>160-165, 202-208
**CANCER B**<br><br>CANCRB	All other malignant neoplasms not in Cancer A or gynecologic ones (including Hodgkin's disease); radiation therapy and chemotherapy encounters where cancer not specified.<br><br>140-159, 170-173, 175, 176, 185-195, 200, 201, V58.0, V58.1, V66.1, V66.2
**CATASTROPHIC CONDITIONS**<br><br>CATAST	Catastrophic conditions, including dissecting aneurysms, cardiac arrest, respiratory arrest, all forms of shock except septic shock; intracranial and subdural haemorrhages.<br><br>Multiple ICD codes
**CHEST PAIN**<br><br>ROAMI	Chest pain, myocardial infarction not specified.<br><br>Misc. 786.5X, V71.7
**CHROIC OBSTRUCTIVE PULMONARY DISORDER**<br><br>COPD	Chronic obstructive pulmonary disorder and some less common respiratory conditions.<br><br>490-496, 500-508, 512, 515, 517-519
**CHRONIC RENAL FAILURE**<br><br>RENAL2	Chronic renal failure, end-stage renal disease, and kidney transplants.<br><br>582, 583, 585-589, 996.81, V42.0xx
**CONGESTIVE HEART FAILURE**<br><br>CHF	Congestive heart failure and some related illnesses.<br><br>Major codes are 425, 428, and miscellaneous (398.91, 402s, 422s, and some 429s, incl. '429')
**DIABETIC KETOACIDOSIS AND**	Diabetic ketoacidosis, with and without coma; hypoglycemic

**RELATED MATABOLIC**<br><br>METAB1	coma; unspecified coma and alteration of consciousness.<br><br>Misc. 250s, 251, 780.0x
**FLUID AND ELECTROLYTE**<br><br>FLaELEC	Typical fluid and electrolyte disorders and dehydration.<br><br>275.2 – 276.9
**FRACTURES AND DISLOCATIONS**<br><br>FXDISLC	All other fractures and dislocations, including pathologic fractures.<br><br>733.1xx, 805-807, 809-819, 822-839, misc. 905, 907, 952
**GASTROINTESTINAL BLEEDING**<br><br>GIBLEED	Gastrointestinal hemorrhage; miscellaneous disorders of stomach and duodenum; diverticulitis; abdominal symptoms, nausea with vomiting; blood in stool.<br><br>530-537, 562, 564, 565, 569, 578, 787, 789, 792.1
**GASTROINTESTINAL, INFLAMMATORY BOWEL DISEASE, AND OBSTRUCTION**<br><br>GIOBSENT	Inflammatory bowel disease and malabsorption; gastrointestinal obstruction; enteritides.<br><br>555-558,560, 568, 579
**GYNECOLOGIC CANCERS**<br><br>GYNECA	Gynecologic malignancies other than ovarian cancer; female breast cancer.<br><br>174, 179-182, 184
**GYNECOLOGY**<br><br>GYNEC1	Non-malignant, non-infectious gynecologic diseases, including benign neoplasms.<br><br>218-221, 256 & multiple miscellaneous codes (including V codes).
**HIP FRACTURE**<br><br>HIPFX	Hip fracture.<br><br>Some 733s, 808, 820, 821, some 905s, 959.6
**INGESTIONS AND BENIGN TUMORS**<br><br>ODaBNCA	Non-gynecologic benign neoplasms; drug overdoses, drug abuse, adverse drug reactions, and poisonings.<br><br>210-217, 222-239, 610, 611; 291, 292, 303-305, 790.3, 796, 960-989, 995.2
**LIVER DISORDERS**<br><br>LIVERDZ	Liver disorders, including hepatitis.<br><br>570-573
**MISCELLANEOUS # 1**<br><br>MISCL1	Miscellaneous conditions not classified previously.<br><br>990-999
**MISCELLANEOUS # 2**<br><br>MSC2a3	External causes of injury; remaining supplemental classification of factors influencing health status and contact with health services.

	Remaining V codes; remaining 790-796; all E codes.
**MISCELLANEOUS # 3**<br><br>MISCL5	Miscellaneous non-cardiac congenital anomalies; miscellaneous symptoms other than fever; miscellaneous tooth and tongue disorders, miscellaneous diagnoses of pain.<br><br>520-529 (tooth & tongue disorders); 740-759, 780 (except for 780.6), 783-785 (if not found elsewhere); 338
**MISCELLANEOUS CARDIAC**<br><br>MISCHRT	Miscellaneous cardiac conditions and congenital heart disease.<br><br>392-405, 745-747
**NON-MALIGNANT HEMATOLOGIC**<br><br>HEMTOL	Hematologic problems other than malignancies.<br><br>273, 280-289, misc 790s, 996.85
**OTHER CARDIAC CONDITIONS**<br><br>HEART2	Diseases of pulmonary circulation and cardiac dysrhythmias.<br><br>415-417, 426, 427, misc. 785s, misc. 996s
**OTHER METABOLIC**<br><br>METAB3	All other endocrine, metabolic and miscellaneous immune disorders (but not including systemic lupus erythematosus or rheumatoid arthritis).<br><br>240-255, 257-272, 274-275.1, 277-279, misc. 790s
**OTHER NEUROLOGICAL**<br><br>NEUMENT	All other neurologic problems and mental disorders (other than drug overdoses); senility.<br><br>290-319, 327-344, 346-389, 781, 797, V71.0
**OTHER RENAL**<br><br>RENAL3	All other renal diseases other than infections.<br><br>Miscellaneous 405s, 591-608, misc. other codes
**OVARIAN AND METASTATIC CANCER**<br><br>CANCRM	Ovarian cancer and metastatic cancer.<br><br>183, 196-199
**PANCREATIC DISORDERS**<br><br>PNCRDZ	Pancreatic disorders.<br><br>577
**PERICARDITIS**<br><br>PERVALV	Pericarditis and valvular heart disease.<br><br>391, 423, 424
**PERINATAL PERIOD**<br><br>PERINTL	All conditions originating in the perinatal period.<br><br>760-779
**PNEUMONIA**<br><br>PNEUM	All forms of pneumonia; empyema; pleurisy; and lung abscess; also includes pulmonary tuberculosis; pulmonary congestion and hypostasis.<br><br>480-487; 510; 511; 513; 011, 012.8; 514
**PREGNANCY**	Pregnancy and related conditions, including circumstances

PRGNCY	related to reproduction and development.<br><br>630-677, V22-V28
**SEIZURES**<br><br>SEIZURE	Seizure disorders.<br><br>345, misc. 780.1-780.4
**SEPSIS**<br><br>SEPSIS	Sepsis, meningitis, septic shock, and major catastrophic infections.<br><br>003.1, 003.21, 027.0, 036-038, 040, 320-326, 422.92, 728.86, 785.4, 785.59, 790.7, 995.92, 9993
**SKIN AND AUTOIMMUNE DISORDERS**<br><br>SKNAUT	SLE, rheumatoid arthritis, skin disorders, & related autoimmune diseases, sialoadenitis<br><br>690-710, 713, 714, 782
**STROKE**<br><br>STROKE	Stroke and post-stroke complications.<br><br>434-438, 997.0x
**URINARY TRACT INFECTIONS**<br><br>UTI	Urinary tract infections, not including pregnancy related ones.<br><br>590, 595, 597, 599, 601, 604, misc. 996s

8 Grouping CPT Codes into CPT Groups

CPT GROUP	DESCRIPTION & INCLUDED CPT CODES
**ANESTHESIA**<br><br>ANES	Head, neck, thorax, intrathoracic, spine and spinal cord, upper and lower abdomen, perineum, pelvis, upper and lower leg, knee and popliteal area, shoulder and axilla, upper arm and elbow, forearm, wrist and hand, radiological procedures, burn excisions or debridement, obstetric, and other.<br><br>00100-01999
**EVALUATION AND MANAGEMENT**<br><br>EM	Office or other outpatient services, hospital observation services, hospital inpatient services, consultations, emergency department services, critical care services, continuing intensive care services, nursing facility services, domiciliary, rest home, or custodial care services, home services, prolonged services, case management services, care plan oversight services, preventive medicine services, special evaluation and management services, and other manual and management services.<br><br>99201-99499
**MEDICINE**<br><br>MED	All other medicine including drug administration, vaccines, toxoids, hydration, therapeutic, prophylactic, and diagnostic injections and infusions, psychiatry, dialysis, ophthalmology,

	contact lens services, spectacle services, medical tests and measurements, analysis, assessment, intervention, evaluative and therapeutic services, diagnostic studies, drug administration, physical medicine and rehabilitation, education and training for patient self-management, special services, procedures and reports, moderate sedation, and home health procedures and services.<br><br>90281-99199, 99500-99602
**PATHOLOGY AND LABORATORY**<br><br>PL	Organ or disease panels, drug testing, therapeutic drug assays, evocative and suppression testing, consultations, urinalysis, chemistry, molecular diagnostics, infectious agent: detection of antibodies, microbiology infectious agent detection, anatomic pathology, cytopathology, cytogenetic studies, and surgical pathology.<br><br>80048-89356
**RADIOLOGY**<br><br>RAD	Diagnostic radiology, diagnostic ultrasound, radiation oncology, and nuclear medicine.<br><br>70010-79999
**SURGERY-AUDITORY SYSTEM**<br><br>SAS	External ear, middle ear, inner ear, and temporal bone, middle fossa approach.<br><br>69000-69979
**SURGERY-CARDIOVASCULAR SYSTEM**<br><br>SCS	Heart and pericardium, and arteries and veins.<br><br>33010-37799
**SURGERY-DIGESTIVE SYSTEM**<br><br>SDS	Lips, vestibule of mouth, tongue and floor of mouth, dentoalveolar structures, palate and uvula, salivary gland and ducts, pharynx, adenoids, and tonsils, esophagus, stomach, intestines, meckel's diverticulum and the messentery, rectum, anus, liver, biliary tract, pancreas, abdomen, and peritoneum, and omentum.<br><br>40490-49999
**SURGERY-EYE AND OCULAR ADNEXA**<br><br>SEOA	Eyeball, anterior segment, posterior segment, ocular adnexa, and conjunctiva.<br><br>65091-68899
**SURGERY-GENITAL SYSTEM**<br><br>SGS	Male: penis, testis, epididymis, tunica vaginalis, scrotum, spermatic cord, seminal vesicles, and prostate; Female: vulva, perineum and introitus, vagina, cervix uteri, corpus uteri, oviduct and ovary, ovary, and in vitro fertilization.<br><br>54000-55899, 56405-58999

**SURGERY-INTEGUMENTARY SYSTEM**<br><br>SIS	Skin, subcutaneous and accessory structures, nails, pilonidal cyst, introduction, repair, destruction, and breast.<br><br>10040-19499
**SURGERY-MATERNITY CARE AND DELIVERY**<br><br>SMCD	Maternity care and delivery.<br><br>59000-59899
**SURGERY-MUSCULOSKELETAL SYSTEM**<br><br>SMS	General, head, neck and thorax, back and flank, spine, humerus and elbow, forearm and wrist, hand and fingers, pelvis and hip joint, femur and knee joint, foot and toes, application of casts and strapping, and endoscopy and arthroscopy.<br><br>20000-29999
**SURGERY-NERVOUS SYSTEM**<br><br>SNS	Skull, meninges, and brain, spine and spinal cord, and extracranial nerves, peripheral nerves, and autonomic nervous system.<br><br>61000-64999
**SURGERY-OTHER**<br><br>SO	Surgery-endocrine system: thyroid gland, and parathyroid, thymus, adrenal glands, pancreas, and carotid body; Surgery-mediastinum and diaphragm; Surgery-operating microscope.<br><br>60000-60699, 39000-39599, 69990
**SURGERY-RESPIRATORY SYSTEM**<br><br>SRS	Nose, accessory sinuses, larynx, trachea and bronchi, and lungs and pleura.<br><br>30000-32999
**SURGERY-URINARY SYSTEM**<br><br>SUS	Kidney, ureter, bladder, and urethra.<br><br>50010-53899

9 Grouping Place of Service

The authors in consultation with hospital physicians grouped the place of service into the following categories.

PLACESVC GROUP	DESCRIPTION & INCLUDED PLACE OF SERVICE CODES
**AMBULANCE**	Ambulance: A vehicle specifically designed, equipped, and staffed for lifesaving and transporting the sick or injured; Ambulatory surgical center: a freestanding facility, other than a physician’s office, where surgical and diagnostic services are provided on an ambulatory basis.
**HOME**	Location, other than a hospital or other facility, where the patient receives care in a private residence.

**INPATIENT HOSPITAL**	A facility, other than psychiatric, that primarlily provides diagnostic, therapeutic, and rehabilitation services by physicians for admitted patients.
**INDEPENDENT LAB**	A laboratory certified to perform diagnostic or clinical tests independent of an institution or a physician's office.
**OFFICE**	Location where the health professional routinely provides health examinations, diagnosis, and treatment of illness or injury on an ambulatory basis.
**OUTPATIENT HOSPITAL**	A portion of a hospital that provides diagnostic, therapeutic (both surgical and nonsurgical), and rehabilitation services to sick or injured persons who do not require hospitalization or institutionalization.
**URGENT CARE**	Urgent care facility: Location whose purpose is to diagnose and treat illness or injury for unscheduled, ambulatory patients seeking immediate medical attention. Emergency room—hospital: A portion of a hospital where emergency diagnosis and treatment of illness or injury is provided.
**OTHER**	All other places of service: Assisted living facility, birthing center, community mental health center, comprehensive inpatient rehabilitation facility, custodial care facility, end-stage renal disease treatment facility, federally qualified health center, group home, hospice, independent clinic, inpatient psychiatric facility, mass immunixation center, military treatment facility, mobile unit, nursing facility, other place of service, psychiatric facility, psychiatric residential treatment center, rural health clinic, skilled nursing facility, public health clinic, unassigned, unknown, tribal 638 provider-based facility.

10 Grouping of Specialty

The authors in consultation with hospital physicians grouped the specialty into the following categories. This grouping was also informed by the specialty definitions provided by the American Medical Association and the Royal College of Physicians and Surgeons of Canada.

SPECIALTY GROUP	DESCRIPTION
**ANESTHESIOLOGY**	Anesthesiology, hyperbaric oxygen treatment, pain management.
**DIAGNOSTIC IMAGING**	Nuclear medicine, nuclear radiology, radiology, diagnostic radiology.
**EMERGENCY**	Emergency medicine, urgent care, intensivist, acute care.
**GENERAL PRACTICE**	Family practice, general practice.
**INTERNAL**	Allergy and immunology, cardiology, cardiology facility, cardiovascular disease, dermatology, dialysis center, endocrinology, metabolism, gastroenterology, geriatrics, gastrointestinal facility, hospital, infectious disease, internal medicine, nephrology, neurology, pulmonary disease,

	rheumatology, sleep medicine, sports medicine.
**LABORATORY**	Laboratory, reference laboratory.
**OBSTETRICS AND GYNECOLOGY**	Gynecological oncology, gynecology, obstetrics, reproductive endocrinology.
**PATHOLOGY**	Pathology, neuropathology
**PEDIATRIC**	Adolescent medicine, neonatology, pediatric allergy, cardiology, endocrinology, gastroenterology, hematology, oncology, infectious disease, nephrology, neurology, otolaryngology, pathology, pulmonology, radiology, rheumatology, surgery, and urology, perinatology.
**REHABILITATION**	Occupational medicine, orthotics and prosthetics, physical medicine and rehabilitation, physical therapy, rehabilitation therapy.
**SURGERY**	Abdominal, ambulatory, cardiovascular, colon and rectal, general, hand, head and neck, maxillofacial, neurological, neurosurgery, ophthalmology, orthopedic, outpatient, plastic, thoracic, trauma, urology, vascular, wound care.
**OTHER**	All other specialties: Acupuncture, ambulance, blood, chiropractic, clinical and social worker, clinical pharmacy, convalescent care, custodial care, dentist, durable medical equipment, genetics, hematology, home, hospice, infusion, marriage and family counseling, mental health, neurospychology, not specified, nutrition, oncology, optomology, other, pharmacy, psychiatry, psychology, podiatry, public health and general medicine, radiation oncology, registered dietician, skilled nursing, speech therapy, transplant.

11 Relationship to Previous Work

Previous work that is relevant for the de-identification of longitudinal medical records consists of research for the de-identification of transactions or the de-identification of trajectories. Transactions consists of *items*, for example, merchandise bought at a store. All of the items in a particular grouping is called an *itemset*. Trajectories appear in the context of de-identifying movements of individuals, for example, the wireless telephone cell towers people pass by as they travel. Below we explain why this previous work cannot be applied directly to our particular problem:

- Methods for the generalization of transactions often employ local recoding [11, 12]. This means that the precision of, say, a claim's date can vary by claim and by patient. For example, one patient may have a claim's date as the quarter and year, and another claim by the same patient may have only the year as the date, whereas another patient's claim date could be generalized to a month and year. This inconsistency in generalization makes a data set difficult to analyze using the most common statistical techniques. An argument has been made that using local recoding in de-identification algorithms creates data analysis difficulties and therefore global recoding is always preferable [13]. In our de-identification we used only global recoding.

- Previous work that looked at trajectories considered sequences of points [14, 15]. An analogy to our context would be if only adjacent claims are allowed to be part of the adversary knowledge. This assumption does not apply and our problem is more complex because the claims do not need to be in a sequence/adjacent. For example, for a power of 3, an adversary may have background on a patient's first, fifteenth, and twentieth claim in the data set.

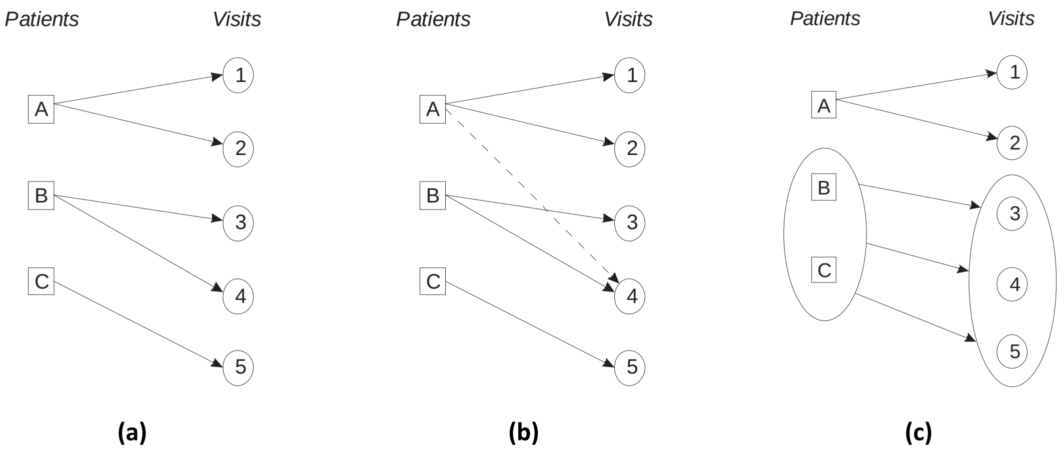

Figure 2: Examples of de-identifications performed on graphs.

- Some previous work looked at the de-identification of graph data [11, 16-19]. These papers either use permutation to alter the graph structure (deleting and inserting edges) and/or they partition the nodes of the graph and their corresponding entities into groups, and only the maps between the groups are revealed. To illustrate permutation and grouping approaches, assume we have a set of 3 patients: A,B and C, and a set of 5 claims: claims 1 and 2 correspond to patient A, 3 and 4 correspond to patient B and 5 to patient C (see panel a in Figure 2).

  A permutation can assign an additional claim to a patient, say patient A to claim 4 (panel b), or remove a claim from the record of a patient. Grouping is illustrated in panel c, where it hides the mappings between the entities and only reports whether two groups have a map between them. In this case we would know that between patients B and C they had claims 3, 4, and 5, but would not know which patient had which claim(s).

  Permutation (whether combined with grouping or not) does not retain the structure of the graph. In [11], the authors use only grouping, thus they preserve the graph structure exactly, however the resulting anonymized data would be locally recoded.

	Wine	Meat	Cream	Strawberries	Sensitive Items
**Bob**	X	X			Viagra
**David**	X	X			
**Ellen**	X	X	X		
**Andrea**		X		X	Pregnancy test
**Claire**			X	X	

Table 6: Example of using generalization and permutation to de-identify transactional data.

- In [20], the authors use generalization and permutation to deal with the problem of attribute disclosure. Their method relies on grouping the transactions with varying sensitive values together, thus forming several “anonymized groups”. Then they publish the quasi-identifiers of each group together with a summary of the sensitive items in the group. In other words, the sensitive attributes are linked to the whole group and not to a particular transaction in the group. Table 6 provides an example of 5 purchase transactions after being anonymized using the method in [20]. Note that the data is divided into 2 groups: the first 3 records form one group and the other 2 form the second group. Each group has one sensitive data associated with it. An adversary knows that the sensitive values correspond to one record in their group but the exact correspondence is hidden.

  Besides, our focus is on protecting against identity disclosure and not attribute disclosure. The above method would lead to significant data distortion and produces data sets that would be difficult to analyze. Furthermore, this method uses the fact that in transaction data, sensitive attributes are rare and that does not apply to our case.

Other research which considered the power of the adversary always assumed that the power is fixed for all patients [2-6]. We have argued that this simplifying assumption may not hold in practice because patients would differ on how easy it is to construct background knowledge about them, and developed a method to model such variation. Some researchers have taken a different approach and suggested that the data custodians should define possible groupings of the items in a transaction to meet certain privacy and utility requirements [21, 22]. For the HHP data set it is not clear how all of the quasi-identifiers can be grouped a priori and how the proposed approach would work with multiple transactions treams (one for each quasi-identifier).

12 References

1. Eguale T, Bartlett G, Tamblyn R. Rare visible disorders / diseases as individually identifiable health information. Proceedings of the American Medical Informatics Association Symposium. 2005.

2. Xu Y, Fung B, Wang K, Fu A, Pei J. Publishing sensitive transactions for itemset utility. Eighth IEEE International Conference on Data Mining. 2008.
3. Xu Y, Wang K, Fu A, Yu P. Anonymizing transaction databases for publication. Conference on Knowledge Discovery and Data Mining. 2008.
4. Terrovitis M, Mamoulis N, Kalnis P. Privacy preserving anonymization of set valued data. Proceedings of the Very Large Databases Endowment, 2008; 1(1):115-125.
5. Liu J, Wang K. Anonymizing transaction data by integrating suppression and generalization. Proceedings of the 14th Pacific-Asia Conference on Knowledge Discovery and Data Mining. 2010.
6. He Y, Naughton J. Anonymization of set-valued data via top-down, local generalization. Very Large Databases (VLDB). 2009.
7. Maggurran A. Measuring Biological Diversity. 2004; MA, USA: Blackwell Publishing.
8. Simpson E. Measurement of Diversity. Nature, 1949; 163:688.
9. Dankar F, El Emam K. A method for evaluating marketer re-identification risk. Proceedings of the 3rd International Workshop on Privacy and Anonymity in the Information Society. 2010.
10. Escobar G, Greene J, Scheirer P, Gardner M, Draper D, Kipnis P. Risk-Adjusting Hospital Inpatient Mortality Using Automated Inpatient, Outpatient, and Laboratory Databases. Medical Care, 2008; 46(3):232-239.
11. Cormode G, Srivastava D, Yu T, Zhang Q. Anonymizing bipartite graph data using safe groupings. Proceedings of rhe Very Large Data Bases Endowment. 2008.
12. Nergiz M, Clifton C, Nergiz A. Multirelational k-anonymity. IEEE Transactions on Knowledge and Data Engineering, 2009; 21(8):1104-1117.
13. El Emam K, Dankar F, Issa R, Jonker E, Amyot D, Cogo E, Corriveau J-P, Walker M, Chowdhury S, Vaillancourt R, Roffey T, Bottomley J. A Globally Optimal k-Anonymity Method for the De-identification of Health Data Journal of the American Medical Informatics Association, 2009; 16(5):670-682.
14. Noman M, Fung B, Debbabi M. Walking in the crowd: Anonymizing trajectory data for pattern analysis. CIKM. 2009.
15. Pensa R, Monreale A, Pinelli F, Pedreschi D. Pattern-preserving k-anonymization of sequences and its application to mobility data mining. PiLBA. 2008.
16. Backstrom L, Dwork C, Kleinberg J. Wherefore are though R3579X ? Anonymized social networks, hidden patterns and structural stenography. WWW. 2007.
17. Hay M, Miklau G, Jensen D, Weis P, Srivastava S. Anonymizing social networks. 2007; University of Massachusetts at Amherst.
18. Zheleva E, Getoor L. Preserving privacy of sensitive relationships in graph data. Privacy, Security, and Trust in KDD. 2007: Springer.
19. Korolova A, Motwani R, Nabar S, Xu Y. Link privacy in social networks. ICDE. 2008.
20. Ghinita G, Tao Y, Kalnis P. On the anonymization of sparse high-dimensional data. IEEE International Conference on Data Engineering. 2008.
21. Loukides G, Gkoulalas-Divanis A, Malin B. COAT: Constraint-based Anonymization of Transactions. Knowledge and Information Systems, 2011; 28:251-282.
22. Loukides G, Gkoulalas-Divanis A, Shao J. Anonymizing transaction data to eliminate sensitive inferences. DEXA. 2010.
